# The Structure, Function and Regulation of Protein Tyrosine Phosphatase Receptor Type J and Its Role in Diseases

**DOI:** 10.3390/cells12010008

**Published:** 2022-12-20

**Authors:** Huiting Li, Peng Zhang, Cencen Liu, Yiwei Wang, Yan Deng, Wei Dong, Yang Yu

**Affiliations:** 1 Key Laboratory of Medical Electrophysiology, Ministry of Education & Medical Electrophysiological Key Laboratory of Sichuan Province, (Collaborative Innovation Center for Prevention of Cardiovascular Diseases), Institute of Cardiovascular Research, Southwest Medical University, Luzhou 646000, China; 2 People’s Hospital of Zhongjiang County, Deyang 618100, China; 3 Department of Human Anatomy and Histoembryology, School of Basic Medical Sciences, Southwest Medical University, Luzhou 646000, China

**Keywords:** protein tyrosine phosphatase receptor type J (PTPRJ), antioncogene, metabolic diseases, neurological disorders, signaling pathway

## Abstract

Protein tyrosine phosphatase receptor type J (PTPRJ), also known as DEP-1, HPTPη, or CD148, belongs to the R3 subfamily of receptor protein tyrosine phosphatases (RPTPs). It was first identified as an antioncogene due to its protein level being significantly downregulated in most epithelial tumors and cancer cell lines (e.g., colon, lung, thyroid, breast, and pancreas). PTPRJ regulates mouse optic nerve projection by inhibiting the phosphorylation of the erythropoietin-producing hepatocellular carcinoma (Eph) receptor and abelson murine leukemia viral oncogene homolog 1 (c-Abl). PTPRJ is crucial for metabolism. Recent studies have demonstrated that PTPRJ dephosphorylates JAK2 at positions Y813 and Y868 to inhibit leptin signaling. Akt is more phosphorylated at the Ser473 and Thr308 sites in *Ptprj*^−/−^ mice, suggesting that PTPRJ may be a novel negative regulator of insulin signaling. PTPRJ also plays an important role in balancing the pro- and anti-osteoclastogenic activity of the M-CSF receptor (M-CSFR), and in maintaining NFATc1 expression during the late stages of osteoclastogenesis to promote bone-resorbing osteoclast (OCL) maturation. Furthermore, multiple receptor tyrosine kinases (RTKs) as substrates of PTPRJ are probably a potential therapeutic target for many types of diseases, such as cancer, neurodegenerative diseases, and metabolic diseases, by inhibiting their phosphorylation activity. In light of the important roles that PTPRJ plays in many diseases, this review summarizes the structural features of the protein, its expression pattern, and the physiological and pathological functions of PTPRJ, to provide new ideas for treating PTPRJ as a potential therapeutic target for related metabolic diseases and cancer.

## 1. Introduction

In eukaryotes, the phosphorylation and dephosphorylation of amino acid residues such as tyrosine and serine play a crucial role in the development of pathophysiology, involving cell metabolism, apoptosis, proliferation, differentiation, and other physiological functions [1,2]. Protein tyrosine kinases (PTKs) and protein tyrosine phosphatases (PTPs) have competing functions that regulate the post-translational modification of tyrosine, which happens quickly, can be reversed, and plays a vital role in ensuring normal physiological processes [3]. Human PTPs consist of 107 members [4] and, depending on the amino acid sequence and substrate specificity, are classified into four categories: (1) classical PTPs, which include receptor-type protein tyrosine phosphatases (RPTPs) and non-receptor-type protein tyrosine phosphatases (nRPTPs); (2) dual-specificity phosphatases (DSPs); (3) low molecular weight phosphatases (LMW-PTPs); and (4) CDC25 class phosphatases [5].

Ten years after the discovery of PTKs, Tonks et al. discovered PTPs in the late 1980s [6]. PTPs are essential cell surface proteins with intracellular tyrosine phosphatase activity and an extracellular structural domain with sequence homology to that of cell adhesion molecules [7]. RPTPs are type I integral membrane proteins that play regulatory roles in a variety of regulatory processes in the body, including cell differentiation, migration, metabolism, adhesion, and cancer development [8]. There are currently 39 human PTPs, divided into 17 subtypes, 22 of which are RPTPs [9]. Based on extracellular homologous sequences and PTP domains, RPTPs were divided into eight subfamilies: R1/R6, R2a, R2b, R3, R4, R5, R7, and R8 [10]. RPTPs are functional counterparts of PTKs, which maintain the dynamic balance of cellular tyrosine phosphorylation by inhibiting the phosphorylation activity of PTKs [11]. Shintani et al. showed that partial RPTPs negatively regulate the activation of RPTKs by dephosphorylating specific tyrosine residues, thereby inhibiting their function [10]. RPTPs have a similar structural composition consisting of an extracellular structural domain, a transmembrane structural domain, and one or two intracellular tyrosine phosphate domains, with the N-segment extracellular structural domain of subfamily members being highly diverse and the intracellular tyrosine phosphate region being highly conserved [12]. There are five R3 RPTPs in humans and mice: protein tyrosine phosphatase receptor type J (PTPRJ), PTPRB, PTPRO, PTPRH, and PTPRQ [13].

**Figure 1:**
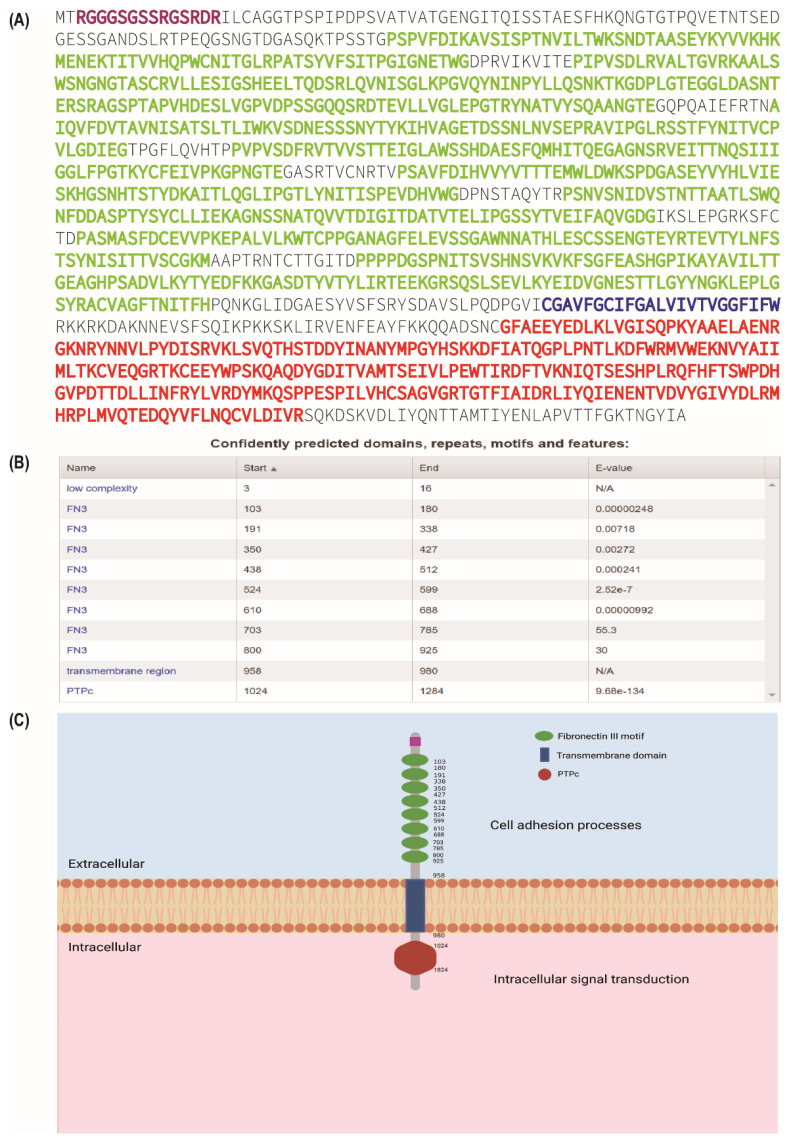
The amino acid sequence, sequence analysis, and structural sketch of the human PTPRJ. (**A**) The amino acid sequence of human PTPRJ was obtained from the NCBI database (http://www.ncbi.nlm.nih.gov/ accessed on 6 December 2022). The amino acid sequences of the eight fibronectin III motifs are highlighted in green, the amino acid sequence of a transmembrane domain is highlighted in dark blue, and the amino acid sequence of the intracellular phosphatase catalytic domain is highlighted in red. (**B**) The predicted domains of PTPRJ were analyzed by SMART (http://smart.embl-heidelberg.de/ accessed on 6 December 2022). (**C**) The domain architecture of the human PTPRJ. The figure was created using BioRender (www.biorender.com accessed on 6 December 2022).

PTPRJ, also known as DEP-1, PTP-eta, HPTPη, or CD 148, is a 220 kDa transmembrane protein consisting of an extracellular domain with eight fibronectin III motifs, a transmembrane structural domain, and an intracellular catalytic domain, which were identified by the Simple Modular Architecture Tool (Figure 1a,b) [14]. Its intracytoplasmic structural domain is involved in intracellular signal transduction pathways, while the extracellular structural domain, which contains eight fibronectin III motifs, may be involved in cell adhesion processes (Figure 1c). PTPRJ was first identified as a tumor suppressor gene that regulates angiogenesis, cell proliferation, and migration through the negative regulation of the activity of various PTKs, including platelet-derived growth factor receptor (PDGFR) [15,16], hepatocyte growth factor receptor (HGFR), epidermal growth factor receptor (EGFR) [17], vascular endothelial growth factor receptor 2 (VEGFR2) [18], and rearranged during transfection (RET) [19]. Therefore, PTPRJ was found to play a role in the advancement of some human malignancies [20,21,22,23,24,25]. PTPRJ expression has been reported to increase with increasing cell density, suggesting a role in cell density–dependent growth inhibition, and is therefore also known as DEP-1 [26]. In addition, PTPRJ could negatively regulate intracellular signaling mediated by a variety of oncogenic RTKs [27,28], suggesting that it may provide a novel therapeutic target for the treatment of several tumors.

PTPRJ is widely expressed in different cell types, including fibroblasts, vascular endothelial cells, vascular smooth muscle cells (VSMCs), epithelial cells, hematopoietic cells, and neurons [16,29,30]. It has been reported that replacement of the PTP structural domain of PTPRJ with enhanced green fluorescent protein (EGFP) sequences in mice resulted in early embryonic death due to vascular deficiency [31], which suggested that the phosphatase activity of PTPRJ plays a prominent role in angiogenesis during embryonic development. Other studies have shown that PTPRJ knockout mice can survive [32,33], and further studies have found that PTPRJ is involved in optic nerve projection, feeding, etc., through the regulation of different PTKs [10].

The *Ptprj* gene encodes the classical PTPRJ as well as another splice variant. A 3193-bp transcript known as sPTPRJ is produced by selectively splicing the PTPRJ mRNA and is substantially shorter than the PTPRJ transcript. There are five type III fibronectin structural domains in the 539aa sPTPRJ protein, which lacks transmembrane and cytoplasmic structural domains as a result of mRNA premature termination during translation [34]. Similar to PTPRJ, sPTPRJ is a protein that is extensively expressed. Adnexal or suspension cells, such as KASUMI cell culture, as well as tumor and normal cell lines, such as 

HUVEC, human umbilical vein endothelial cells, express sPTPRJ [35].

Further results demonstrated that PTPRJ modulates microglia phagocytosis and migration through the negative regulation of Fyn tyrosine kinase, one of the non-receptor-type tyrosine kinases of the Src family [33]. Recent studies have shown that microRNA-204-5p promotes pre-eclampsia serum-induced endothelial cell injury in human umbilical veins through regulation of the PTPRJ/Notch axis [36].

Here, we reviewed and compiled the most current knowledge on the structure, expression, and regulation of PTPRJ and its contribution to cellular phagocytosis, axonal projection, cancer, and metabolic diseases.

## 2. The Relationship between PTPRJ and Cancer

Recent studies, through whole-exome sequencing of the human cancer genome, have shown that PTPRJ is mutated in a variety of cancers [24]. PTPRJ plays a crucial role in tumor pathogenesis, and its expression is significantly reduced in some malignant tumors, such as human meningioma and breast, pancreatic, thyroid, colon, lung, and cervical carcinoma cancers [37]. Frequent deletion of PTPRJ, allelic imbalance in loss of heterozygosity (LOH), and missense mutations have been identified in human colon, lung, and breast cancers [38]. Thus, missense polymorphisms are often considered one of the mechanisms affecting the function of the PTPRJ molecule [39,40]. PTPRJ overexpression in diverse cancer cell lines was shown to exert a negative regulatory effect on cell proliferation, migration, differentiation, and cell adhesion, as well as on transformation, and is therefore considered to be a tumor suppressor [24]. The mechanism of the anti-proliferative effect of PTPRJ may be attributed to the inhibition of the phosphorylation of various PTKs, such as the FMS-like tyrosine kinase 3 (FLT3), EGFR, PDGFR, VEGFR2, HGFR, and extracellular signal-regulated kinase 1/2 (ERK 1/2) (Table 1) [15,18,41,42,43,44]. In addition, recent studies have shown that PTPRJ acts to inhibit cancer cell proliferation by regulating the expression of mRNA and DNA methylation and is regulated by a number of microRNA, such as miR-328, miR-155, etc. [45,46].

### 2.1. Association between PTPRJ and Gastric Cancer

**Figure 2:**
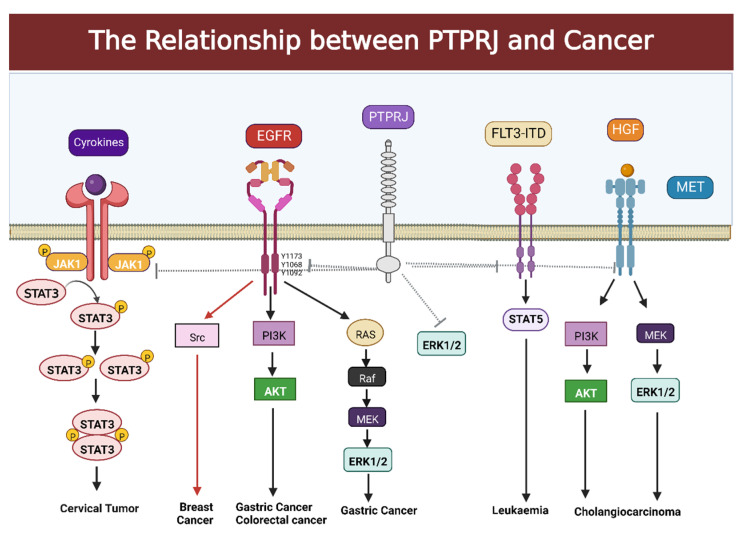
The relationship between PTPRJ and cancer. In cervical cancer, PTPRJ inhibits the activation of the JAK1/STAT3 pathway by decreasing the phosphorylation levels of JAK1 and STAT3. We have indicated with red arrows that PTPRJ cooperates with EGFR to increase the activation of Src-dependent signaling pathways downstream of aggressive breast cancer cells to promote tumor cell invasion and metastasis. PTPRJ negatively regulates the AKT signaling pathway in gastric and colorectal cancers. In addition, PTPRJ can also inhibit the MEK/ERK pathway in gastric cancer. In leukemia, PTPRJ inhibits FLT3 and its downstream STAT5 signaling pathway. In cholangiocarcinomas, PTPRJ has been shown to recognize MET as a substrate to inhibit PI3K/Akt and MEK/ERK downstream signaling pathways. This figure was created using BioRender (www.biorender.com accessed on 6 December 2022).

Gastric cancer, which is the fourth most common cancer worldwide, poses a threat to human health, and tumor formation mechanisms and possible therapeutic targets have long been a hot research topic. Sun et al. confirmed that higher expression of the PTPRJ is associated with longer overall survival times, mature cell differentiation, and reduced vascular invasion in patients with gastric cancer [45]. However, the knockdown or knockout of PTPRJ increased the ability of gastric cancer cells to grow and metastasize in vitro and in vivo, and suppression of PTPRJ expression resulted in poor clinical characteristics and a poor prognosis in patients with gastric cancer [45]. Mechanistically, PTPRJ substantially inhibited the downstream PI3K/AKT and MEK/ERK pathways and negatively affected the phosphorylation of several EGFR tyrosine residues, including Y1173, Y1068, and Y1092 (Figure 2) [45]. Targets become hyperphosphorylated in the absence of a functional PTPRJ, which results in aberrant growth. Furthermore, the 3′UTR region of the PTPRJ gene has high DNA methylation levels, and there is a strong association between these levels and PTPRJ expression, indicating that DNA methylation may be a significant regulator of PTPRJ expression levels in gastric cancer [45]. Therefore, it is hypothesized that PTPRJ may be a potential predictor of prognosis in gastric cancer and that the study of PTPRJ as a potential therapeutic target for gastric cancer will be a future research direction.

### 2.2. Regulation of PTPRJ in Hepatocellular Carcinoma

Hepatocellular carcinoma (HCC) is the most common type of liver cancer [50], representing approximately 90% of all liver cancers [57]. Patients with advanced HCC have an overall 5 year survival rate of less than 5% due to intra- and extra-hepatic metastases, early invasion of blood vessels, and recurrence [46]. Paduano et al. demonstrated the direct regulation of PTPRJ mRNA levels by miR-328 through binding to its 3′-UTR in various cancer cells [37,58]. Luo et al. showed that the level of migration and invasive ability of HCC cells may be regulated through the interaction of miR-328 and/or PTPRJ [47]. The deletion of PTPRJ significantly attenuated the effects of miR-328 inhibitors on cell migration and invasion [47]. Thus, the interaction of miR-328 with PTPRJ caused the miR-328-dependent proliferation of epithelial cancer cells.

### 2.3. PTPRJ and Its Relationship with Cholangiocarcinoma

Cholangiocarcinoma is a malignant tumor occurring in the bile duct system, the exact etiology of which is not known. Studies have shown that both p38 and cellular mesenchymal-epithelial transition factor (c-Met) promote the proliferation and invasion of human cholangiocarcinoma cells [48], while p38 plays a crucial role in maintaining high c-Met activity, and this action is mediated by the inhibition of c-Met dephosphorylation. When c-Met activation decreases, then the two classical downstream pathways of c-Met, PI3K/Akt and MEK/ERK, are downregulated. However, P38 inhibits downregulation of Akt and ERK phosphorylation levels, thereby promoting their activity, proliferation, and invasion in cholangiocarcinoma cells, and conversely, PTPRJ has been shown to recognize the receptor PTK Met as a substrate [42,48,59,60]. Further studies revealed that PTPRJ is involved in the hepatocyte growth factor (HGF) tyrosine kinase receptor c-Met, which affects the pro-tumor capacity of p38 [42,48]. Mechanically, when c-Met binds to HGF, the catalytic structural domain’s tyr1235 and tyr1234 residues are phosphorylated, and so this activates the downstream PI3K/Akt and MEK/ERK pathways, which are important in cell proliferation and migration [49,61]. The above studies indicated that the proliferation and invasion of cholangiocarcinoma cells are closely related to the relationship between the upregulation of Met and the downregulation of PTPRJ.

### 2.4. Regulation of PTPRJ in Colorectal Cancer

Colorectal cancer (CRC) is a malignant lesion occurring in the colonic mucosal epithelium and is a common malignancy of the gastrointestinal tract [62]. PTPRJ frequently shows a loss of heterozygosity in human colon cancers [39]. PTPRJ is also a candidate gene for the mouse colon cancer suppressor 1 (Scc1) locus [39]. In addition, PTPRJ regulates the function of tight junctions (TJs) in human colonic Caco-2 cell lines, and its expression is regulated by peroxisome proliferator–activated receptor-γ (PPARγ) [63]. PTPRJ was identified as a direct target of miR-155 in colorectal cancer, and the ectopic expression of PTPRJ was found to inhibit cell growth, migration, and invasiveness in the colorectal cancer cell line HCT116, revealing a key role for the miR-155/PTPRJ/AKT axis in cell proliferation and migration [23]. Mechanistically, miR-155 binds to the 3′-UTR region of PTPRJ, thereby negatively regulating PTPRJ and resulting in reduced mRNA levels and protein levels of PTPRJ. Overexpression of PTPRJ levels can eliminate the activation of cell proliferation and the AKT signaling pathway by miR-155 [23]. A number of food nutrients, such as butyrate, green tea, and apple polyphenols, have been shown to upregulate endogenous PTPRJ mRNA transcription and PTPRJ protein expression and may act as chemoprotective foods against colon carcinogenesis [64].

### 2.5. PTPRJ and Its Relationship with Thyroid Cancer

Approximately 1% of all systemic malignancies are thyroid carcinomas (THCA), the most prevalent malignant tumor of the thyroid gland, and their exact etiology is unknown [65]. Current studies have shown that PTPRJ expression levels are negatively regulated by tumor cell transformation. Restoration of the expression of PTPRJ can inhibit the formation of a malignant phenotype [66]. Iuliano et al. discovered that thyroid carcinoma patients had higher rates of the PTPRJ genotypes homozygous for the Gln276Pro, Arg326Gln polymorphisms, and Asp872 allele than in healthy people [50]. Furthermore, the PTPRJ loss of heterozygosity (LOH) was found to be more common in the thyroid carcinomas of heterozygotes for Gln276Pro and Arg326Gln than in homozygotes [50]. Thus, these results demonstrated that the genotype of PTPRJ influences susceptibility to thyroid cancer and that deletion of the allele of PTPRJ is associated with the development of thyroid cancer [50].

### 2.6. PTPRJ and Its Relationship with Breast Cancer

Breast cancer is one of the cancerous tumors that represent a serious threat to women’s health, and its incidence is increasing year by year with a trend toward youthfulness [67]. No definitive cause of breast cancer has been identified to date, but many high-risk factors associated with breast cancer have been identified. It has been demonstrated that PTPRJ is frequently lost in breast cancer, that expression is significantly lower than in normal breast tissue, and that low levels of expression are associated with poorer overall survival [67]. Another study has shown that the PTPRJ gene is a protective haploid in breast cancer [68].

However, it is worth noting that studies have shown that PTPRJ expression is higher in highly invasive breast cancer cells (MDA-MB-231, Hs578T, and BT-549) than in non-transformed or less invasive breast cancer cell lines (MCF-7, T47D, SK-BR3, and MCF10A) [51], contrary to the significantly reduced expression of PTPRJ in many malignancies we mentioned earlier. It is suggested that PTPRJ is associated with increased recurrence and reduced survival in breast cancer patients. Related experiments have found that catalytic expression of PTPRJ is required in conjunction with EGFR activity, which is associated with an increase in the number of its cell protrusions and an enhanced capacity for cell migration and invasion [51]. Further, Spring et al. demonstrated that PTPRJ collaborates with EGFR to increase the activation of the downstream Src-dependent signaling pathway in aggressive breast cancer cells in order to promote tumor cell invasion and metastasis (Figure 2) [51].

### 2.7. PTPRJ and Its Relationship with Leukemia

Leukemia is a malignancy of the hematopoietic system. Previous studies have identified an important role in hematopoietic development for the FLT3 gene [53]. FLT3 is regulated by phosphorylation or dephosphorylation, and an siRNA screen of 20 RPTPs and PTPs identified that the loss of PTPRJ expression results in enhanced FLT3 activation, suggesting that PTPRJ can negatively regulate the phosphorylation of FLT3 [41]. In cells expressing an internal tandem duplication (ITD) mutation in FLT3/ITD, reversible oxidation of reactive oxygen species (ROS) leads to PTPRJ inactivation, and PTPRJ inactivation contributes to FLT3-ITD-mediated cell transformation (Figure 2) [52,54].

### 2.8. PTPRJ and Its Relationship with Cervical Tumor

The fourth most prevalent type of cancer in the world is cervical cancer, which, despite prevention through screening and vaccinations, is still one of the leading causes of cancer-related death among women [69]. Similar to other cancers, PTPRJ is significantly downregulated in human cervical tumors [55,70]. It was shown that PTPRJ downregulation significantly increased cell viability, growth, and migration rates and the transition from G1 to S phase, and this phenomenon was rescued by the overexpression of PTPRJ in cervical cancer C33A cells [55]. The mechanism is related to how PTPRJ inhibits the Janus kinase 1 (JAK1)/Signal transducer and activator of transcription 3 (STAT3) pathway’s activation by decreasing the phosphorylation levels of JAK1 and STAT3 (Figure 2). PTPRJ may be a suitable target for gene therapy in cervical cancer because it also controls the expression of STAT3 downstream factors such as cyclin D, Bax, VEGF, and MMP2 [71]. Cisplatin is one of the most effective drugs for the treatment of cervical cancer. In a recent study, Roychowdhury et al. found enhanced expression of PTPRJ transcript levels in cisplatin-tolerant human cervical cancer SiHa cells [70].

### 2.9. PTPRJ in Other Cancers

Meningioma is one of the most common tumors of the central nervous system (CNS), accounting for approximately 15–20% of tumors. The LOH of the PTPRJ gene and loss of PTPRJ protein expression were identified in a subpopulation of human meningiomas [40]. The negative regulation of PDGF receptor signaling and the positive regulation of adhesion signaling by PTPRJ cooperatively inhibited the motility of meningioma cells and may have suppressed tumor invasiveness [40]. Petermann and co-investigators also argued for a reduction in cell matrix adhesion in PTPRJ-deficient cells, as well as an enhancement in cell motility [40]. In addition, the process of meningioma development is closely related to the deletion of neurofibromatosis type 2 (NF2), which is encoded by the NF2 tumor suppressor gene located on chromosome 22q 12 [1]. Loss of NF2 protein (Merlin) expression due to mutations in the *NF2* gene is one of the most common causes of benign brain tumors (including schwannomas and meningiomas) [56]. Meningioma cells are inhibited by PTPRJ [40], and deletion of PTPRJ increases meningioma cell motility in vitro and invasive growth in an orthotopic xenograft model. Cre/lox-mediated knockout of *NF2* resulted in a 4-fold increased rate of meningioma formation within a year in Ptprj knockout mice compared to wild-type (WT) littermates [1]. This suggests that the deletion of PTPRJ and Merlin contributes to the development of meningiomas; however, the exact mechanisms are still unknown. Additionally, deletion of PTPRJ promotes NF2-dependent meningioma development [1]. Moreover, PTPRJ is a potential tumor suppressor gene for non-small cell lung cancer (NSCC) [72] and non-Hodgkin’s lymphoma (NHL) [73]. Carlos Aya-Bonilla et al. have shown that PTPRJ inactivation may be a common mechanism of lymphangiogenesis in these NHL subtypes and that PTPRJ haplotypes may contribute to NHL susceptibility by affecting PTPRJ activation in such B-cell lymphomas [74].

Another report showed a significantly higher upregulation of PTPRJ in glioblastoma multiforme (GBM), which is contrary to previous reports, suggesting that the potential “double-edged sword” concept of PTPRJ in the pathogenesis of tumors and the GBM-specific cancer-promoting function of PTPRJ need further investigation [75]. The expression of sPTPRJ mRNA is markedly increased in high-grade glioma tissues. The highest grade of gliomas, known as glioblastomas, are highly vascularized brain tumors whose development is mostly dependent on tumor-associated angiogenesis. It is suggested that sPTPRJ may function as an angiogenic factor in combination with the activation of angiogenesis and cell migration by sPTPRJ expression in HUVECs and the downregulation of endothelial cell adhesion molecule expression by sPTPRJ. However, the mechanism by which it promotes angiogenesis is unknown. The researchers speculate that sPTPRJ may affect glioblastoma cells by producing new tumor blood vessels [25]. Given the role of PTPRJ in tumorigenesis, it has the potential to be developed as a prognostic marker for a variety of clinical tumors to facilitate the development of new diagnostic/prognostic or therapeutic strategies.

## 3. Contribution of PTPRJ to the Regulation of Metabolism

Along with rapid social development, the standard of living has gradually increased. Changing patterns of nutrient preference and exercise have led to a growing problem of obesity, which not only increases the risk of hypertension, hyperlipidemia, and other metabolic diseases but also poses a threat to mental health. How obesity is tackled and the mechanisms by which it develops are of great importance in today’s society. Studies have indicated that various RPTPs, such as insulin receptor substrate 1 (IRS-1), RPTPα, RPTP-γ, RPTPκ, RPTP-ε, RPTP-β/ζ, and so on, play vital roles in insulin signaling and secretion [76]. In addition, all R3 RPTP family molecules can dephosphorylate the insulin receptor (IR) in HEK 293 cells [74]. Thus, an exploration of the inhibitors of RPTPs, or targeted drug design with extracellular structures that mimic RPTPs, offers new prospects for the treatment of these metabolic diseases.

### 3.1. PTPRJ and Its Relationship with Insulin Resistance and Type 2 Diabetes

**Figure 3:**
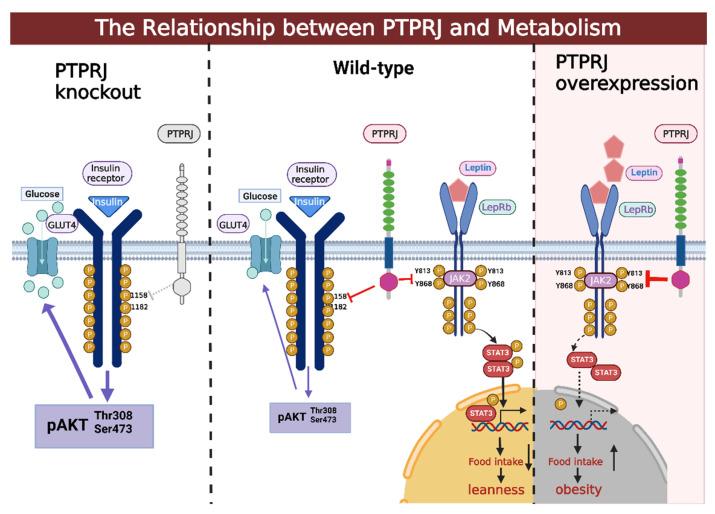
The relationship between PTPRJ and metabolism LepRb dimerizes as a result of leptin binding to the receptor molecule. Following JAK2 autophosphorylation, certain tyrosine residues in LepRb’s intracellular tail are activated. As a result, downstream signaling proteins like STAT3 are successively phosphorylated and activated. When STAT3 is phosphorylated, it moves to the nucleus, where it binds to promoter sites to regulate the transcription of numerous genes. PTPRJ inhibits leptin signaling by dephosphorylating Y813 and Y868 of JAK2. PTPRJ may be a novel negative regulator of insulin signaling, and in *Ptprj*^−/−^ mice, the downstream signaling molecule Akt is more phosphorylated at the Ser473 and Thr308 sites. This ultimately leads to improved insulin signaling through GLUT to promote glucose uptake. This figure was created using BioRender (www.biorender.com accessed on 6 December 2022).

Leptin and insulin are significant molecules that play an important role in maintaining metabolic homeostasis. When insulin is deficient or IR is abnormal, a large amount of glucose cannot enter the cells, is stored in the body, and is eliminated in the urine, the cornerstones of insulin resistance and type 2 diabetes. Activation of the insulin receptor will initiate a signaling cascade, leading to phosphorylation of the IR itself, insulin receptor substrates, and downstream signaling components, which is antagonized by some PTPs, such as protein tyrosine phosphatase 1B (PTP1B), PTPRJ, LAR, and TC-PTP [32,77]. Lower blood glucose is the result of the cell transferring the glucose transporter 4 (GLUT4) to the cell membrane of adipose tissue and skeletal muscle for glucose uptake, which is mediated by the signaling molecule Akt. The higher phosphorylation of the downstream signaling molecule Akt at the Ser473 and Thr308 sites, which ultimately results in facilitated glucose uptake via the GLUT, is one of the ways that Krüger et al. demonstrated that insulin signaling is improved in PTPRJ-deficient (*Ptprj*^−/−^) mice (Figure 3) [32]. Imbalances in insulin-signaling components are evident in diabetes and insulin resistance and can lead to impaired glucose utilization and hyperglycemia [78]. Insulin resistance also plays an important role in the development of the metabolic syndrome and type 2 diabetes [74]. Researchers have previously demonstrated that PTPRJ is involved in the regulation of insulin signaling, and that such a regulatory function is achieved by attenuating IR activation (Figure 3) [79]. It was found that *Ptprj*^−/−^ mice showed increased phosphorylation and glucose uptake by their skeletal muscle cells, suggesting that PTPRJ may be a new negative regulator of insulin signaling [32](Table 2).

### 3.2. PTPRJ and Its Relationship with Obesity

Leptin-activated leptin receptor (LepRb) signals act on hypothalamic neurons to reduce food intake and increase energy expenditure [80,81]. Leptin binds to LepRb, causing the receptor molecule to dimerize. Although LepRb is not a PTK and is not directly regulated by PTPs, it can form complexes with the non-receptor tyrosine kinase JAK2. This autophosphorylation of JAK2 then activates specific tyrosine residues in the intracellular tail of LepRb, sequentially phosphorylating and activating downstream signaling proteins like STAT3. Phosphorylated STAT3 (pSTAT3) relocates to the nucleus, where it binds to promoter sites to control the transcriptional activity of several genes [82] (Figure 3). Furthermore, the phosphorylation level of JAK2 is regulated by many PTPs, such as PTP1B, T cell protein tyrosine phosphatase (TC-PTP), SHP2, PTPN9, and PTPRJ [83]. Previous reports have indicated that PTPRJ was found to inhibit leptin signaling [82]. PTPRJ is widely expressed in the body, including in the hypothalamus, and negatively regulates leptin signaling by dephosphorylating Y813 and Y868 of JAK2 [10](Table 2). *Ptprj*^−/−^ mice were able to survive and reproduce without significant abnormalities [10]. Leptin signaling is enhanced in *Ptprj*^−/−^ mice. In addition, *Ptprj*^−/−^ mice on a normal diet (ND) had a significantly reduced body weight and food intake compared with WT mice [10]. Thus, PTPRJ induction is a contributor to the development of leptin resistance, and suppression of PTPRJ may be a possible strategy for ameliorating obesity (Figure 3).

**Table 2:** Mechanism of protein tyrosine phosphatase receptor type J (PTPRJ) in metabolic disorders.

Metabolic disorders	Mechanism	Cellular/Molecular Function	Reference
Obesity	Dephosphorylating Y813 and Y868 of JAK2	JAK2/STAT3	[10]
Type 2 diabetes	PTPRJ as a negative regulator of insulin signaling.	AKT	[32]

## 4. Contributions of PTPRJ to Axon Projection and Neurological Disorders

### 4.1. The Function of PTPRJ in Visual Topographic Map Formation

PTPRJ and PTPRO are expressed in developing mouse retinal ganglion cells (RGCs) [84]. It was shown in chicks that PTPRO, but not PTPRJ, regulates the projection of retinal axons to the tectum (the avian homolog of the mammalian superior colliculus (SC)) via the dephosphorylation of the Eph receptor [85]. It is possible that when PTPRJ is knocked out, its function is complemented by other R3 family molecules, such as PTPRO. However, further research indicated that the topographic mapping of retinal axons in the optic chisam (OC) and the superior colliculus (SC) were abnormal only in *Ptprj*^−/−^ mice, not in *Ptpro*^−/−^ mice. Further investigation demonstrated that PTPRJ, but not PTPRO, plays a key role in the projection of the optic nerve by regulating the tyrosine phosphorylation of Eph and Abl [84]. This difference may be attributable to species differences: the amino acid sequences of the intracellular region (ICR) of mouse PTPRJ and chick PTPRJ are only 78% identical [86], while the amino acid sequence of PTPRO has 93% identity [84]. In addition, the expression level of Ptprj in the mouse retina was significantly elevated compared to Ptpro [84]. Therefore, PTPRJ seems to play a prominent role in visual projection.

### 4.2. The Relationship between PTPRJ and Neurological Disorders

Autism spectrum disorders (ASDs) are a cluster of behaviorally defined neurodevelopmental conditions that are considered to be one of the most complex neuropsychiatric disorders. They carry heritable features that are of a lifetime nature and have a significant impact on social communication as well as social activities. Expression Quantitative Trait Loci (eQTL) analysis of homogeneous blocks of subclasses A1 and B1 revealed associated polymorphisms with dysregulation of the important autism candidate gene PTPRJ-JAK2 [87].

In addition to the expression of PTPRJ in neurons as well as peripheral macrophages, PTPRJ is expressed in microglia. Microglia express a wide range of phagocytic receptors, such as scavenger receptors, Fc receptors, and related proteins, including PTPRJ, toll-like receptors (TLRs), and others, which play an important role in the phagocytosis of infectious particles, apoptotic cells, neurons, and pathological protein aggregates, such as Aβ in Alzheimer’s disease [88]. Furthermore, when stimulated with lipopolysaccharide (LPS), the expression of MARCO, TLR2, and PTPRJ was upregulated in microglia [88]. Together, these experiments suggested that PTPRJ may have an essential function in microglia-mediated neuroinflammation-associated diseases, and further studies could be focused on the involvement of PTPRJ in inflammation-related neurodegenerative diseases.

## 5. The Role of PTPRJ in Osteogenesis

PTPRJ promotes osteoclast (OCL) maturation by balancing the pro- and anti-osteoclastogenic activities of M-CSFR and by maintaining NFATc1 expression during late osteoclastogenesis, thereby preventing the key osteoclastogenic transcription factor NFATc1 from ubiquitination and degradation [87]. The absence of PTPRJ increases the ubiquitination of NFATc1 and reduces the expression of NFATc1 during late osteoclastogenesis, thereby inhibiting OCL maturation [89]. By dephosphorylating the M-CSF receptor (M-CSFR) and the Cbl family of ubiquitin ligases (Cbl), PTPRJ promotes OCL maturation [89].

## 6. The Relationship between PTPRJ and Platelets

PTPRJ is the most abundant RPTP in platelets, playing a vital role in the regulation of platelet function [90]. The surface of human platelets contains approximately 2800 copies of PTPRJ, with little variation between individuals [91]. It has been shown that PTPRJ deficiency can improve the degree of thrombocytopenia [92]. Src, Lyn, and Fyn are three Src family kinases (SFKs) that are crucial for platelet activation as well as for megakaryocyte (MK) development and platelet production [93,94]. C-terminal Src kinase (Csk), which phosphorylates a conserved tyrosine in the C-terminal tail of platelet SFKs, inhibits the SFK activity. PTPRJ, which dephosphorylates the same residue, activates them [93]. Inherited thrombocytopenias (ITs) are a large, heterogeneous group of diseases characterized by abnormally low platelet counts that may lead to a tendency to bleed. Although the genetic reasons for ITs are becoming better understood, variants of unknown origin afflict approximately 50% of patients with familial thrombocytopenia [95,96]. Small-sized platelets, spontaneous bleeding, and decreased platelet responses to the GPVI agonists collagen and convulxin have all been observed to be symptoms of the illness induced by PTPRJ mutations [97]. The reduced activation of Src family kinases may be the cause of these platelet functional abnormalities [97].

## 7. The Relationship between PTPRJ and Immune Function

**Figure 4:**
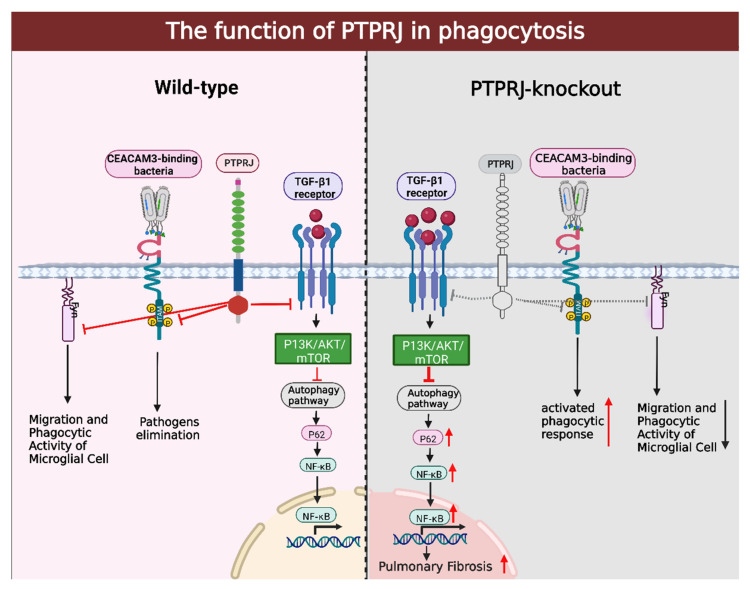
The function of PTPRJ in phagocytosis. The TGF-β1-induced PI3K/Akt/mTOR signaling pathway could be enhanced by PTPRJ deficiency, blocking the autophagic pathway and resulting in p62 accumulation. In contrast, overexpression of PTPRJ can decrease the accumulation of p62 and have an anti-fibrotic effect by suppressing p62-dependent NF-κb-mediated production of pro-fibrotic gene expression. PTPRJ alters and decreases CEACAM3 phosphorylation, thereby negatively regulating CEACAM3-mediated phagocytosis and limiting the potential inflammatory response. PTPRJ positively regulates microglia migration and phagocytosis through dephosphorylation of microglia at the Tyr42 site of the Fyn tyrosine kinase. This figure was created using BioRender (www.biorender.com accessed on 6 December 2022).

PTPRJ is widely expressed on and regulates a variety of immune cells such as T lymphocytes, B lymphocytes, macrophages, and granulocytes to achieve a role in the immune response or related diseases [98,99]. Tsoyi and other researchers found that PTPRJ is downregulated in clinical cases of Idiopathic Pulmonary Fibrosis (IPF) and that PTPRJ downregulation modulates the profibrotic response. PTPRJ deficiency would upregulate the TGF-β1-induced PI3K/Akt/mTOR signaling pathway, inhibiting the autophagic pathway and leading to p62 accumulation. Conversely, overexpression of PTPRJ can reduce the accumulation of p62, thus exerting an anti-fibrotic effect by inhibiting p62-dependent nuclear factor-kappaB (NF-κB)-mediated pro-fibrotic gene expression (Figure 4). Some extracellular proteins such as SDC2 can bind to PTPRJ and activate its activity, reducing fibrosis levels in vivo as well as in vitro in lung fibrosis models, providing new ideas in the treatment of IPF [30].

Carcinoembryonic antigen-related cell adhesion molecule 3 (CEACAM3) is encoded by the CEACAM3 gene, which is found only in granulocytes of higher primates and plays a role in mediating cellular phagocytosis in defense against pathogenic infections such as *Neisseria gonorrhoeae*. Recent studies by Goob et al. have shown that PTPRJ alters and decreases CEACAM3 phosphorylation without altering c-Src phosphorylation levels, possibly by acting directly on CEACAM3, thereby negatively regulating CEACAM3-mediated phagocytosis and limiting the potential inflammatory response. Through CEACAM3-mediated granulocyte phagocytic response, on the one hand, the organism can be protected from pathogenic microorganisms; on the other hand, an over-activated phagocytic response will damage cells or tissues, especially PTPRJ, which is particularly important for the negative regulation of CEACAM3, which exerts a certain protective effect on the body while clearing pathogens (Figure 4) [100].

In addition, microglia, as the main phagocytic cells in the brain and one of the main immune defenses of the CNS, will migrate to the lesion site and be activated in the event of intracerebral lesions or CNS dysfunction. Activated microglia are not only morphologically altered but also play a role in CNS homeostasis by releasing various inflammatory mediators and phagocytosis of apoptotic cells and myelin debris. In PTPRJ-deficient BV-2 cells (a murine microglial cell line), their migratory capacity was reduced by 50% compared to controls, and in the same in vivo experiments, the number of microglia in PTPRJ-deficient brains was not significantly altered, yet their migratory capacity was significantly reduced. Previous studies have shown that the functional regulation of microglia by PTPRJ appears to be achieved through the regulation of SFKs [98]. Schneble et al. demonstrated in vitro and in vivo that PTPRJ positively regulates microglia migration and phagocytosis, and this facilitation is achieved in part by dephosphorylation of the Ty420 site of Fyn to inhibit Fyn kinase activity (Figure 4) [33].

## 8. PTPRJ May Be a Potential Therapeutic Target

Dysregulated angiogenesis is linked to pathological conditions, such as ischemic heart disease, as well as cancer, diabetes, or chronic inflammation [101]. Studies have shown that PTPRJ is abundantly expressed in vascular endothelial cells and negatively regulates endothelial cell proliferation [102,103]. This suggests that inhibition of endothelial cell proliferation by PTPRJ may be a potential therapeutic target for the treatment and amelioration of angiogenic dysregulation-related diseases. Based on this, Takahashi et al. designed Ab1, a monoclonal antibody targeting the human PTPRJ ectodomain sequence, which has high specificity and high affinity for PTPRJ in endothelial cells. Ab1 can enhance PTPRJ-mediated signaling. Researchers found that bivalent (intact) Ab1 inhibited vascular endothelial cell growth and blocked blood vessel formation in the mouse cornea [104].

Studies have shown that PTPRJ is identified as a regulator of the C-terminal tyrosine phosphorylation of EGFR [17]. It was shown that inhibition of the phosphatase activity of PTPRJ by dimerization prevents PTPRJ from accessing its RTK substrates. Homodimerization of PTPRJ is modulated by specific transmembrane (TM) residues, and disruption of these interactions destabilizes the homodimerization of full-length PTPRJ in the cell. Subsequently reducing the phosphorylation of the known PTPRJ substrate EGFR and other downstream signaling effectors, ultimately inhibiting the EGFR-driven cellular phenotype [44]. Thus, the interaction of homodimers of PTPRJ could be a new approach to treating certain cancers. Targeted drug interventions for levels of PTPs in disease have been widely described, and these drugs have shown good experimental results in disease models; however, to date, there are no clinically approved drugs for PTPRJ, and further research is needed on drug interventions for PTPRJ levels for the clinical treatment of metabolic diseases or cancer.

## 9. Concluding Remarks and Future Directions

The aim of this review is to highlight the PTPRJ mechanisms explored in cancer, metabolic diseases, axon guidance, and neuroinflammation. PTPRJ was first identified as a tumor suppressor gene and has been found to be decreasingly expressed in a variety of cancers, including gastric cancer, HCC, colorectal cancer, and cervical cancer. However, a significantly higher upregulation of PTPRJ has been reported in GBM, suggesting that the regulatory role of PTPRJ may be more complex, and further research is needed to explore the role of PTPRJ in different cancers and at different stages of cancer.

Currently, PTP inhibitors have been explored in clinical trials [105,106,107,108], such as one trial examining the role of inhibitors of PTP1B in metabolic diseases [109], but inhibitors of PTPRJ have not yet been identified, which may be an indication for future research. PTPRJ plays an important role in axonal projection, angiogenesis, cell proliferation, neuroinflammation, metabolism, and cancers by dephosphorylating various members of the RTK signaling pathway; therefore, PTPRJ may provide a promising therapeutic target in inflammation and cancers, as well as in other relevant diseases.

## Figures and Tables

**Table 1:** Mechanism of protein tyrosine phosphatase receptor type J (PTPRJ) in cancer.

Cancer	Mechanism	Cellular/Molecular Function	Reference
Gastric Cancer	Methylation in the 3′UTR region of the PTPRJ gene	EGFR, PI3K/AKT, and MEK/ERK pathways	[45]
Hepatocellular Carcinoma	MiR-328 acts directly on the 3′-UTR of PTPRJ	Not identified	[47]
Cholangiocarcinoma	c-Met dephosphorylation	PI3K/Akt and MEK/ERK	[42,48,49]
Colorectal Cancer	LOH of PTPRJ	AKT	[23]
Thyroid Cancer	LOH of PTPRJ	Not identified	[50]
Breast Cancer	Deletion and/or mutation	SRC, EGFP	[51]
Leukemia	PTPRJ oxidation	FLT3	[52,53,54]
Cervical tumor tissue	Decreasing the phosphorylation levels of JAK1 and STAT3	JAK1/STAT3	[55]
Human Meningiomas	LOH of PTPRJ	PDGF	[56]
